# Cardiovascular Risk and Quality of Life in Elderly People with Mild Thyroid Hormone Deficiency

**DOI:** 10.3389/fendo.2014.00153

**Published:** 2014-10-07

**Authors:** Sara Tognini, Giuseppe Pasqualetti, Valeria Calsolaro, Antonio Polini, Nadia Caraccio, Fabio Monzani

**Affiliations:** ^1^Geriatrics Unit, Department of Clinical and Experimental Medicine, University of Pisa, Pisa, Italy

**Keywords:** elderly, subclinical hypothyroidism, quality of life, cardiovascular risk, cardiovascular events, heart failure, ischemic heart disease, mortality

## Abstract

Subclinical hypothyroidism (sHT) is a common condition in the general population, the prevalence increases with age, especially in women. An association between sHT and increased coronary heart disease (CHD) and heart failure (HF) risk and mortality has been described. However, this association is far to be established in older people (>65 years), especially in the oldest old (>85 years). Individuals with sHT may experience symptoms that resemble those observed in the overt form of the disease, leading to an impaired quality of life (QoL). Although very old people are frequently frail and potentially more susceptible to the effects of a disease, few studies were designed to assess the effect of sHT on QoL in this subset of population. Interestingly, the serum TSH concentration curve of general population has a skewed distribution with a “tail” toward higher values, which is amplified with aging. Thus, the diagnosis of sHT and the interpretation of its potential effects on CV function and QoL in older people may be a challenge for the clinician. Giving these premises, we reviewed the English scientific literature available on National Library of Medicine (www.pubmed.com) since 1980 regarding hypothyroidism, sHT, elderly, cardiovascular risk, CHD or HF events and mortality, health-related QoL, and LT4 therapy. Consistent results among large prospective cohort studies suggest an age-independent relationship between sHT and HF progression, while an impact of sHT on CHD events and mortality is essentially reported in young adults (aged below 65–70 years) with long-lasting disease. Scanty data are available on QoL of older people with sHT (>65 years) and, generally, no significant alterations are described.

## Introduction

Subclinical hypothyroidism (sHT), defined as serum TSH concentration above the upper limit of the reference range in the face of normal free T_4_ (FT_4_) and free T_3_ (FT_3_) levels, is a common condition in older people, especially among women (1–3). This biochemical condition encompasses several pathological entities, mainly represented by chronic autoimmune thyroiditis. sHT is often associated to symptoms that resemble those of overt hypothyroidism, although to a lesser extent thus, the expression “mild thyroid impairment” or “mild thyroid hormone deficiency” would be more appropriate for defining such a condition (1). Nonetheless, the term sHT is recognized worldwide and will be utilized in the present review, too. In the last decades, an association between sHT and increased cardiovascular (CV) risk has been described, although depending on the degree of TSH elevation (1, 4, 5). However, this relationship in older individuals is far to be established (6–9). Although very old people are frequently frail and potentially more susceptible to the effects of a disease, few studies were designed to assess the effect of sHT on quality of life (QoL) in this subset of population. Moreover, the serum TSH concentration curve of general population has a skewed distribution with a “tail” toward higher values, which is amplified with aging (6, 7). Thus, the diagnosis of sHT and the interpretation of its potential effects on CV function and QoL in older people may be a challenge for the clinician. Thus, better understanding of these topics could, at least partially, resolve the question of whether treating older people, especially the oldest old (aged > 85 years), with sHT is appropriate, especially in case of slight elevation (from >4 to <10 mIU/l) of serum TSH value. Given these premises, we reviewed the English scientific literature available on National Library of Medicine (www.pubmed.com) since 1980 regarding hypothyroidism, sHT, elderly, CV risk, coronary heart disease (CHD) or heart failure (HF) events and mortality, health-related QoL, and LT4 therapy.

## Thyroid Function and Aging

The relationship between thyroid function and aging has been hypothesized more than almost two decades ago (8). Several clinical studies confirmed an age-dependent decrease of thyroid function including iodine uptake and thyroid hormone production (9). However, it should be underlined that direct age-related changes need to be distinguished from the actual alterations induced by thyroid diseases or non-thyroidal illness. In this setting, conflicting results still exist regarding the serum TSH reference range and its modification with aging between earlier reports (mainly case-control or cross-sectional) and recent large naturalistic studies (3, 7, 8, 10–14). Given that sHT is essentially recognized as abnormal serum TSH elevation, the definition of a worldwide recognized age-related reference range is clearly warranted.

In the study by Mariotti et al. (8), healthy centenarians showed a relatively low prevalence of sHT (7%) in the face of reduced serum FT_3_ levels as compared to either young adults or older people (>65 years). Interestingly, a progressive decrease of serum TSH levels from young adults up to older people and centenarians was observed. The study raised the question whether the decreased FT_3_ and TSH values represented an adaptive mechanism to reduced metabolic homeostasis or a protective condition in aging. Moreover, the age-related changes of thyroid status should be distinguished from the “no-thyroidal illness syndrome” (frequently observed in older people), in which serum FT_3_ decreases while FT_4_ (within the normal range of healthy adults) and reverse T_3_ (above the normal range of healthy adults) increase. Indeed, the low serum T_3_ of non-thyroidal illness syndrome reflects a catabolic state and is associated with a lower physical function and poorer prognosis (15, 16). At partial odds with the earlier reports (8, 14), more recent population-based and cross-sectional studies (6, 7) showed a progressive shift of the normal serum TSH range toward higher values from healthy young individuals up to centenarians. A possible interpretation of this finding could be the presence of low bioactive isoforms of circulating TSH (17). Since TSH bioactivity is related to post-translational modifications such as sialylation, sulfation, and glycosylation that could be affected by the aging process, TSH immunoreactivity cannot be assumed to represent TSH bioactivity at all. Thus, it is possible that differences in TSH bioactivity as well as alterations of renal or liver function could account for some of the discrepancies of literature in older people. Unfortunately, current assays for serum TSH measurement cannot distinguish different TSH isoforms and we can only speculate on this finding without certain evidences.

Overall, however, available data suggest that aging is associated with a certain degree of down-regulation of the hypothalamus pituitary–thyroid-peripheral axis, although the clinical significance of such condition and the effectiveness of LT_4_ replacement therapy are far to be established, especially in the elderly (9, 18). Indeed, thyroid hormones (TH) are implicated in maintaining and integrating metabolic homeostasis at multiple levels, notably not only in the hypothalamus but also in peripheral tissues. In detail, TH may affect cell membrane composition, inflammatory response, and stem cell renewal. Interestingly, data from both human beings and animal models showed a negative correlation between serum TH value and longevity. Bowers et al. (19) recently reviewed the available literature on the putative TH-related mechanisms that could be implicated in modulating lifespan of mammals, including human beings. They argued that constraints on TH signaling at certain life stages, notably during old age, could be advantageous for optimal aging. Accordingly, Rozing et al. reported that the offspring of nonagenarian siblings presented a lower thyroidal sensitivity to TSH and a paradoxical beneficial cardio-metabolic profile as compared to their partners (20). Moreover, better survival and performance status has been described in very old people (>85 years) with mild thyroid failure (10, 21–23).

As a whole, these findings suggest that a certain degree of down-regulation of thyroid function may exert a beneficial effect during aging and might represent a protective factor in the oldest old population. It should be underlined, however, that most data were obtained in animal models, mainly rodents, and a parallel with human beings should be cautiously interpreted.

## Epidemiology of sHT

Human aging, especially in women, is associated with increased prevalence of circulating anti-thyroid antibodies and mild to full-blown thyroid failure (2, 3, 10, 18, 24, 25). In this setting, however, the sensitivity of the TSH assay utilized in the study and the cut-off value used for defining sHT, which ranged from >3.0 to >7.0 mIU/l (26, 27) should be taken into account. Moreover, giving the high prevalence of anti-thyroid antibodies observed in individuals with circulating TSH value above 2.5 mIU/l, such cut-off has been also proposed for defining sHT (28, 29). Thus, it is not surprising that the reported prevalence of sHT ranges widely from 1.3 to 21% (24–27, 30–32). Besides the different cut-off values, another factor responsible for the wide variability of sHT prevalence is the heterogeneity of the population analyzed in each trial, which differs in relation to age, lifestyle, comorbidity, treatments, and ethnicity as well as the different degrees of iodine intake (18). Indeed, in people with sufficient iodine intake, a higher prevalence of both thyroid autoimmunity and hypo is generally observed as compared to individuals living in iodine insufficient areas (33). Accordingly, in the Wickham survey, the prevalence of sHT was 7.5% but rose from 7.5 to 17.4% while considering older women (>75 years) with positive serum anti-thyroid antibody titers (28). In the Colorado STUDY, 21% of women and 16% of men older than 74 years suffered from mild thyroid deficiency (10). Interestingly, the prevalence of sHT decreased by 2% for patients older than 75 years and by 5% for those older than 90 years while using an age-specific reference range (7, 12, 24). Another crucial aspect to keep in mind is the rate of progression from mild to overt hypothyroidism. Indeed, a significant number of subjects experienced no progression and even normalization of serum TSH value (34, 35).

In conclusion, the prevalence and the clinical impact of sHT as reported by scientific literature always should be interpreted in relation to the demographic, ethnic, and lifestyle characteristics of the studied population. Moreover, the actual presence of sHT should be confirmed by at least two measurements, with sufficient interval, of serum TSH level.

## Thyroid Hormones and the Cardiovascular System

Subclinical hypothyroidism might be interpreted as an intermediate alteration between full-blown thyroid failure and euthyroidism. Thus, changes of CV system induced by sHT qualitatively resemble those observed in overt hypothyroidism even if less evident. Since TH influence each structure of the heart and its conducting system, the relationship between thyroid dysfunction and CV disease goes well beyond the risk of atherosclerosis and atrial fibrillation (1, 36, 37). TH, mainly FT_3_, regulate myosin heavy chain genes, which encode for the two contractile protein isoforms of the thick filament of the cardiomyocyte (38–41). TH also induce expression of the sarcoplasmatic reticulum Ca2-ATPase and reduce the expression of its inhibitor, phospholamban (42). Accordingly, reduced heart output along with prolonged isovolumic relaxation phase, leading to an early cardiac impairment, has been described in hypothyroidism (43). Many studies documented similar alteration also in sHT patients (44–46). Systolic function impairment with increased pre-ejection/ejection time ratio and mean aortic acceleration has been shown in sHT patients (47–49). Decreased cardiac preload along with increased after load, leading to a reduction of cardiac output, was confirmed by magnetic resonance study (50). Interestingly, similar functional modifications are described during the aging process, thus it is not surprising that sHT may worsen the age-related cardiac process resulting in increased risk of HF progression and events (51). Moreover, cardiac alterations associated with sHT are generally reversed after restoration of euthyroidism by l-thyroxine replacement (44, 47, 52).

Thyroid hormones also affect endothelium and smooth muscle cell function, leading to reduced vascular tone (38). Several mechanisms for T_3_-mediated vascular relaxation have been reported (37, 38, 53, 54). As stated by Klein et al., T_3_ induces *in vitro* relaxation of smooth muscle cells independently of NO production, suggesting a direct effect of the hormone (38). Dardano et al. showed that, in patients monitored for differentiated thyroid cancer, the administration of recombinant human TSH acutely impaired endothelium-dependent vasodilation, possibly through the induction of low-grade inflammation and reduced NO availability by oxidative stress, suggesting a possible role of TSH per se in endothelial dysfunction (55). Moreover, sympathetic and adrenal activation may contribute to the development of arterial hypertension in hypothyroid individuals (56–58).

Overall, these findings suggest that sHT affects CV system by complex mechanisms involving both myocardium and vasculature. These effects are partially similar to those typically observed in the aging process, making difficult to distinguish CV changes of aging from those directly belonging to sHT. Both the degree of serum TSH increase and its long-lasting effect have been claimed to concur to the development of sHT effects at tissue level (59, 60). Thus, exposition to minute thyroid hormone deficiency may, over time, be associated with increased risk of CV diseases. However, the actual burden of mild thyroid failure on the CV system of older people remains to be established and still is widely debated (1, 38).

## Heart Failure and sHT in the Elderly

Heart failure, defined as an impaired ability of the ventricle to fill with or eject blood, represents a common clinical condition with increasing prevalence in the last decades of life (61, 62). More than 20 million people suffer from HF worldwide (63), and its prevalence and incidence are increasing mostly because of increasing lifespan, and also because of increased prevalence of risk factors (diabetes mellitus, systemic hypertension, dyslipidemia) and improved survival rates from other types of CV diseases (64). In 2011, HF was the most common reason for hospitalization for individuals older than 85 years and the second most common for those aged 65–85 years (65). Since sHT has been described to be associated with conditions that may concur to the development of HF (66–68), several prospective studies have been designed to evaluate the possible relationship between mild thyroid dysfunction and HF with discordant results (66, 69–71). As above described, the effect of TH deficiency on CV system is partially similar to that typically observed in the aging process (interstitial fibrosis, myocyte loss, cardiac remodeling). However, few studies directly examined the relationship between sHT and HF in elderly people (70–75). Rodondi et al. studied 3044 adults older than 65 years and initially free of HF over a mean 12-year follow-up (70). An increased risk of HF was observed in subjects with TSH above 10.0 mIU/l with HF incidence of 41.7/1000 vs 22.9/1000 person per year in euthyroid individuals (*p* = 0.01). Interestingly, no increased incidence was observed in subjects with serum TSH level between 4.5 and 9.9 mIU/l, even after stratification by TSH concentration below or above 6.9 mIU/l. Accordingly, among 5316 older adults (aged 70–82 years) an increased HF risk (HR 3.0, 95%CI: 1.12–8.11) was observed only in individuals with serum TSH value above 10.0 mIU/l after 3.2 years of follow-up (51). Nonetheless, in a previous study on community-dwelling older people (2730 subjects aged 70–79 years with a follow-up of 4 years) those with serum TSH level between 7.0 and 10.0 mIU/l showed an increased risk of HF (HR 2.58, 95% CI: 1.19–5.60) but not of other CV events or mortality (72). Moreover, mild elevated TSH (<10.0 mIU/l) represented a negative prognostic factor for increased cardiac and all-cause mortality in hospitalized patients (3121 patients, mean age 61.1 years) with acute HF events (73).

At odds with these results, in a study performed in 5888 community-dwelling individuals aged over 65 years, those with sHT did not show an increased HF risk, even for TSH values above 10.0 mIU/l. Moreover, in a time-varying model, in which thyroid status was updated with subsequent TSH measurements, there was no association between sHT and incident HF (74). Similarly, patients older than 60 years with serum TSH value above 5.0 but less than 8.0 mIU/l did not show an increased risk of hospitalization for HF (HR 1.23, 95% CI: 0.96–1.56) (75). The lack of an association between mild thyroid failure and HF was also reported by a very recent prospective study in subjects aged over 65 years (76). Possible explanations of these conflicting results may be the different ethnic compositions, mean age, and comorbidity of the study populations as well as the different degrees of serum TSH elevation and length of follow-up. Moreover, since in many individuals TSH normalizes over time (15–65% over follow-up periods going from 1 to 6 years), a possible fluctuation of thyroid dysfunction over time might have contributed to the negative findings of some studies, especially in case of single serum TSH determination (35).

In conclusion, most studies showed an increased risk of HF progression and events in sHT patients, although with different strengths of association and, generally, for serum TSH value above 10.0 mIU/l (51, 70–72). Accordingly, a recent meta-analysis of 6 large, prospective studies on community-dwelling older individuals confirmed that sHT patients have an age-independent increased rate of HF, although only for serum TSH value above 10.0 mIU/l (77). Finally, although small case-control studies, mainly performed in young adults, have shown that restoration of euthyroidism by LT_4_ therapy is able to improve indexes of cardiac function (47, 52); no large, randomized, controlled trials have been performed to date to evaluate the efficacy of LT_4_ replacement in preventing HF progression and events.

## Coronary Heart Disease and sHT in the Elderly

Conflicting data still exist regarding the association between sHT and CHD; some studies reporting increased risk (4, 5, 78), some decreased (76), and some the lack of any association (72, 79–83). Since inconsistent data were especially obtained in the elderly (Table 1) the risk of CHD in sHT patients has been suggested to be confined to young adults (<65 years). Nonetheless, most reports did not accurately explore the possible age-related differences in CHD risk, even in case the elderly represented a large share of the enrolled population.

**Table 1:** **Most representative prospective or cross-sectional studies on subclinical hypothyroidism and cardiovascular risk**.

Reference	Study design	Study population	Participants (sHT)	Age (years)	TSH (mIU/l)	Follow-up (years)	Analyzed endpoint	Outcome
Hak et al. (84)	Cross-sectional	Postmenopausal women	1149 (124)	96.0 ± 7.9	>4.00	NA	CVE	OR 2.3 (CI 1.3–4.2)
Parle et al. (83)	Prospective	Community dwelling	1120 (94)	>60	>5.00	10	CVM	HR 0.9 (CI 0.6–1.3)
Lindeman et al. (85)	Cross-sectional	Community dwelling	755 (112)	74.1 ± 8.2	NA	NA	CVE	*p* = 0.0007 (TSH > 10.0)
Imaizumi et al. (78)	Prospective	Community dwelling	2650 (257)	62.7 ± 11.1	7.16 ± 4.82	12.2	ACM, CVE	HR 1.9 (CI 1.1–3.2), in men only OR 2.5 (CI 1.1–5.5)
Gussekloo et al. (23)	Prospective	Community dwelling	502 (30)	>85	>4.8	4	ACM	HR 0.76 (0.62–0.92)
van den Beld et al. (22)	Prospective	Community dwelling	359 (6)	77.8 (73–94)	>4.3	4	Survival	NS
Cappola et al. (80)	Prospective	Community dwelling	3135 (496)	72.7 ± 5.6	6.67 ± 2.54	13	CVM, CVE	HR 1.14 (CI 0.91–1.43), HR 1.04(CI 0.87–1.23)
Iervasi et al. (73)	Prospective	Hospitalized cardiac patients	2113 (208)	61.1 (60.7–61.5)	6.7 (6.01–7.44)	2.7	ACM, CVM	HR 2.0 (CI 1.33–3.04), HR 2.4 (CI 1.36–4.21)
Bauer et al.(86)	Prospective	Community dwelling	438 (37)	71.5 ± 5.0	>5.50	11.9	Survival	HR 1.23 (CI 0.55–2.74)
Razvi et al.(5)	Prospective	Community dwelling	2376 (97)	49.9 ± 17.9	6.00–15.00	20	CVE, CVM	HR 1.76 (CI 1.15–2.71), HR 1.79 (1.02–3.56) 1
McQuade et al. (87)	Retrospective	Community dwelling	6240 (1396)	EU: 54.6 ± 12.7, Mild sHT: 57.1 ± 13.3, Moderate sHT: 58.9 ± 13.6	Mild sHT: 3.1–6.0, Moderate sHT: 6.1–10.0	8	ACM	*p* = 0.026 in moderate sHT people aged > 65 years
de Jongh et al. (88)	Prospective	Community dwelling	1219 (34)	>65	6.89 (5.65–9.59)	10.7	ACM, CVM	HR 0.9 (CI 0.58–1.42), HR 0.5 (CI 0.20–1.49)
Waring et al. (89)	Prospective	Community dwelling	1503 (116)	74	4.79–10.00	8.3	ACM, CVM	HR 1.01 (CI 0.7–1.4), HR 1.28 (CI 0.8–2.1)
Hyland et al. (74)	Prospective	Community dwelling	4863 (697)	73.4 ± 5.7	6.7 ± 2.6	10	CVE, CVM	HR 1.37 (CI 1.0–1.87), HR 0.89 (CI 0.64–1.48)
LeGrys et al. (90)^a^	Prospective	Postmenopausal women	3663 (282)	65–79	> 4.69	>5	CVE: *TSH 4.69*–*6.99, TSH* > *7.00, TSH 4.69*–*6.99, TSH* > *7.00*	*Age 65*–*70:* HR 0.99 (CI 0.42–2.35), HR 1.11 (CI 0.51–2.39); *Age 71*–*79:* HR 0.53 (CI 0.13–2.26), HR 1.27 (CI 0.44–3.69)
Rhee et al. (91)	Prospective	Community dwelling	2570 (691)	sHT: 59.2 ± 19.2	6.3 (5.3–8.72)	14.3	ACM	Pre-existing HF: HR 1.77 (CI 1.19–2.64), No pre-existing HF: HR 0.97 (CI 0.84–1.12)
Ceresini et al. (92)	Prospective	Community dwelling	951 (29)	> 65	>4.68	6	CVM	HR 0.50 (CI 0.10–2.55)
Yeap et al. (82)	Prospective	Community dwelling men	3885 (416)	70–89	> 4.00	6.4 ± 1.5	ACM	HR 1.06 (CI 0.86–1.32)
Frey et al. (69)	Prospective	Hospitalized cardiac patients	1032 (34)	sHT 62 ± 12, EU 67 ± 13	5.29 (4.02–9.38)	3.08 (1.5–3.58)	ACM	HR 0.96 (CI 0.52–1.77)
Perez et al. (75)^b^	Prospective	Community dwelling	4987 (237)	>60	6.40 (5.60–8.00)	2.73	CVM, ACM	HR 1.46 (CI 1.16–1.84), HR 1.36 (CI 1.03–1.76)

*^a^First-time incident myocardial infarction*.

*^b^TSH value lower than 8.00 mIU/l but no distinction between sHT and overt hypothyroidism. Age and TSH value are reported as mean ± SD or median (confidence interval or range)*.

*sHT, subclinical hypothyroidism; EU, euthyroidism; HR, hazard ratio; OR, odds ratio; ACM, all-cause mortality; CVM, cardiovascular mortality; CVE, cardiovascular events; NS, not significant; NA, not available*.

In this setting, a recent large meta-analysis showed no evidence of a gradient of CHD risk while stratifying data by age (93). Moreover, in the Rotterdam study [1149 postmenopausal women aged 69.0 ± 7.5 years (mean ± SD)] sHT was associated with a greater age-adjusted prevalence of aortic atherosclerosis [odds ratio, 1.7 (95% CI 1.1–2.6)] and myocardial infarction [MI, odds ratio, 2.3 (95% CI 1.3–4.0)]. Additional adjustment for body mass index, lipoprotein profile, systemic blood pressure, and smoking status, as well as the exclusion of women taking beta-blockers, did not affect these estimates (84). Similarly, Lindeman et al. documented a significantly higher prevalence of CHD in patients older than 65 years with elevated serum TSH (>10.0 mIU/l) as compared to those with normal TSH value (85). Conversely, most naturalistic studies did not find any association between sHT and increased CHD events or mortality among older people (86–90, 92), even if one documented increased all-cause mortality (81). The Women’s Health Initiative survey (90) examined the association between sHT and MI by measuring serum TSH and FT_4_ in 736 women with incident MI as compared to 2974 randomly selected individuals. After 5–10 years of follow-up, the Hazard ratio for MI was 0.99 (95% CI: 0.67–1.46) for women with serum TSH between 4.69 and 6.99 mIU/l, and 1.19 (95% CI: 0.72–1.96) for those with TSH ≥ 7.0 mIU/l (90). It should be underlined, however, that no information about the use of thyroid medication was available beyond 3 years of follow-up and thyroid function testing was performed only at the beginning. However, Hyland et al., in a prospective study on 4863 older people (> 65 years), in which thyroid status was updated with subsequent TSH measurements, did not find any association between persistent or transient sHT and incident CHD (HR 1.37, 95% CI: 1.00–1.87 and HR 1.00, 95% CI: 0.68–1.49, respectively) or CV death (HR 0.97, 95% CI: 0.64–1.48 and HR 0.99, 95% CI: 0.70–1.39, respectively). Additional analysis stratified by the degree of serum TSH elevation (4.5–6.9; 7.0–9.9, and 10.0–19.9 mIU/l) confirmed the negative findings (74). Another prospective study on 1587 community-dwelling older men (> 65 years) did not find any association between sHT and CV events or total mortality notwithstanding the rate of TSH elevation, but serum TSH higher than 10.0 mIU/l was detected only in eight men (89). Moreover, a cross-sectional study with subgroup analyses by age confirmed that increased CHD risk was present only in younger sHT participants (<50 years old) (94). Accordingly, a meta-analysis of five studies demonstrated that sHT is associated with increased CHD prevalence and events only in young adults (aged < 65 years), in whom an increased incidence of CHD was observed also for serum TSH value lower than 10.0 mIU/l (95). Interestingly, a very large, recent meta-analysis clearly demonstrated that, in age- and sex-adjusted analyses, the risk of CHD events and mortality increases with rising serum TSH level, reaching the statistical significance only in patients with TSH values above 10 mIU/l regardless of patients’ age (93).

In summary, the negative effect of sHT on CHD events and mortality appears well established in subjects younger than 65 years but less evident in older people. The inconsistency in results among studies in elderly patients may be due either to the duration of tissue exposure to sHT or of follow-up, as well as to the presence or not of comorbidity (pre-existing CV or other chronic diseases). The most representative meta-analyses on the association between sHT and CHD risk are reported in Table 2.

**Table 2:** **Most representative meta-analyses on the relationship between subclinical hypothyroidism and coronary heart disease**.

Reference	Number of studies	Study population (subjects with sHT)	Age	Endpoint	Outcome	
					Whole cohort	Subjects older than 65 years
Singh et al. (97)	6	11,495 (935)	58–75	CHD events	RR 1.18 (CI 1.02–1.37)	NA
				CVM	RR 1.11 (CI 0.99–1.25)	
Haentjens et al. (102)	9	13,329 (290)	50–85	CVM	HR 1.22 (95% CI 0.96–1.57)	NA
Ochs et al. (66)	10	14,449 (1491)	46–85	CHD events	HR 1.20 (95% CI 0.97–1.49)	<80 and HR 1.06 (95% CI 0.91–1.24)
						>80 and HR 0.47 (95% CI 0.11–1.90)
				CVM	HR 1.18 (95% CI 0.98–1.42)	<80 and HR 1.12 (95% CI 0.99–1.28)
						>80 and HR 0.55 (95% CI 0.24–1.25)
Razvi et al. (95)	15	29,022 (2531)	42–85	CHD events	HR 1.27 (95% CI 0.95–1.22)	HR 1.02 (95% CI 0.85–1.22)
				CVM	HR 1.09 (95% CI 0.84–1.41)	HR 0.85 (95% CI 0.56–1.29)
Rodondi et al. (93)	11	55,287 (3450)	46–85	CHD events	HR 1.18 (95% CI 0.99–1.42)	<80 and HR 1.20 (95% CI 0.95–1.51)
						>80 and HR 1.30 (95% CI 0.93–1.82)
				CVM	HR 1.09 (95% CI 0.96–1.24)	<80 and HR 1.32 (95% CI 1.08–1.62)
						>80 and HR 1.01 (95% CI 0.62–1.63)

*sHT, subclinical hypothyroidism; CHD, coronary heart disease; CVM, cardiovascular mortality; HR, hazard ratio; NA, not available*.

## Cerebrovascular Disease and sHT

Subclinical hypothyroidism is postulated to increase stroke risk via atherogenic changes associated with abnormal thyroid function. However, the direct relationship of sHT with subsequent stroke is poorly studied. Some naturalistic studies suggest that sHT is not associated to cerebral ischemic events, and a better outcome of patients affected by sHT with respect to those without thyroid failure has been reported (72, 98, 99). In detail, a large naturalistic study (2730 individuals aged 70 to 79 years) with 4-year follow-up data showed that sHT was not associated with a higher prevalence of either CV events (including stroke) or total mortality (80). Schultz et al. (99) prospectively evaluated 609 community-dwelling individuals older than 50 years showing no increase of incident stroke among sHT subjects also after adjusting data by the degree of serum TSH elevation. These data are partially at odds with those from the Women’s Health Initiative Observational Study (98), in which the potential relationship between incident ischemic stroke and severity of sHT was prospectively assessed in postmenopausal women (639 ischemic stroke cases and 2927 randomly selected sub-cohort members with 7 years of follow-up). The adjusted hazard ratio from weighted Cox models resulted modestly elevated (1.22, 95% CI, 0.73, 2.05) only for sHT women with serum TSH value higher than 7.00 mIU/l. However, giving the wide range of 95% CI, the data appeared not sufficiently consistent to suggest a clear-cut association between sHT and ischemic stroke, in otherwise healthy postmenopausal women. In this setting, it is noteworthy that among 756 patients with acute ischemic stroke those with sHT at admission were more likely to show favorable functional outcomes (100).

In conclusion, available data suggest that sHT has no impact on cerebral ischemic events or the effect is too small to be detected in naturalistic studies. Preconditioning before stroke along with reduced response to stress could represent the possible protective mechanism exerted by sHT in the outcome of patients with stroke. More studies are clearly warranted to better address this topic with dedicated epidemiological assessment.

## sHT and CV Mortality

Although an association between sHT and a greater all-cause and CV mortality (73, 87, 101, 102) has been generally documented in individuals with underlying high CV risk (patients at high risk for or who had recent cardiac events), it has not always been observed in general population (23, 72, 79, 80, 88, 89). Indeed, a large population-based, longitudinal study of CHD and stroke in subjects older than 65 years failed to support the hypothesis of an association between unrecognized sHT and increased CV events or mortality (80). Although no difference in the association between sHT and mortality among patients with and without underlying CV disease was detected by Rodondi et al. (93), in a more recent meta-analysis, pre-existing congestive HF significantly worsened the relationship between sHT and HF events (77). It is plausible that subpopulations with congestive HF may be predisposed to cardiac morbidity and mortality associated with sHT. In agreement with this hypothesis, only sHT patients (aged 59.2 ± 19.2 years, mean ± SD) with congestive HF showed a greater risk of all-cause mortality compared to euthyroid individuals (HR 1.77, 95% CI: 1.19–2.64) (91). Moreover, Iervasi et al., among 3308 patients attending a Cardiology Clinic, documented an increased risk of cardiac death in those with sHT (HR 2.40, 95% CI: 1.36–4.21), after 32 months of follow-up (73). There are few clinical studies evaluating the effects of hormone replacement in sHT subjects and none aimed at determining the impact of LT_4_ treatment on total mortality, especially in older people. Previous research in this area has shown contradictory results, with some randomized, controlled trials showing an improvement of both dyslipidemia and surrogate endpoints of atherosclerosis (36, 103, 104) not documented by previous, uncontrolled studies (105–108).

In conclusion, the relationship between mild thyroid failure and increased risk of cardiac and all-cause mortality is far from being established and widely accepted, especially in the elderly. A negative effect of sHT on HF progression and events nonetheless has been documented in older individuals with underlying high CV risk. Large, randomized intervention studies are currently ongoing in older people with sHT and will share new insight in this still unresolved issue (109).

## sHT and QoL in the Elderly

Quality of life has been defined by the World Health Organization as “an individual’s perception of their position in life in the context of the culture and value system in which they live, and in relation to their goals, expectations, standards and concerns” (110). Thus, patients are the best judges of their own perceived QoL. Measuring health status is imperative, especially in case of chronic diseases in which long-term survival is not at risk and the goal of treatment is to maintain patients symptom-free and living in the community. Symptoms and QoL in hypothyroidism were assessed by a large number of instruments, which can be broadly divided into two categories: generic and disease specific (111); most used generic tools, validated in thyroid failure and used in various thyroid disorders, are the Short Form36 (SF-36), the Nottingham Health Profile (NHP), and the General Health Questionnaire (GHQ) (111–115). Various validated thyroid-specific questionnaires were also developed: the Chronic Thyroid Questionnaire (CTQ), the Underactive-Thyroid-Dependent Quality of Life Questionnaire (ThyDQoL). and the Underactive Thyroid Treatment Satisfaction Questionnaire (ThyTSQ) (116–119). It is well known that overt hypothyroidism slows metabolism, with classical symptoms as tiredness, fatigue, and weight gain, and induces neuromuscular symptoms and cognitive problems (1). Moreover, overt hypothyroidism is associated with high psychiatric morbidity, in particular depression and paranoid disorders (120, 121). Thus, it is not surprising that overt thyroid failure is associated with impaired QoL (112, 116, 117, 122). It is generally recognized that sHT encompasses symptoms comparable to those observed in the overt form of the disease, although to a lesser extent (10). Mild thyroid failure has been associated with mood and cognitive alterations as well as neuromuscular abnormalities (48, 120, 121, 123, 124). To date, however, the literature regarding the effects of sHT on QoL is sparse and inconclusive (114, 115, 117, 125, 126). The largest naturalistic study suggested that sHT does not significantly affect health-related QoL (114, 115), while other studies carried out in sHT patients referring to endocrine out-patient clinics documented a significant decrease of QoL (125, 127). Scanty data are also available on the effect of LT4 replacement therapy on QoL (107, 128–130). Some studies did not find a significant modification of health-related QoL (107, 128), while others documented an improvement by restoring euthyroidism (129–131). As in adult population, some observational studies suggested an association between sHT and mood disorders in older people that could worsen QoL (120, 132). In detail, in 323 subjects older than 60 years, Chueire et al. showed that sHT increases the risk for depression (OR 4.9, 95% CI 2.8–8.6) (120). Moreover, of 163 sHT older patients (>65 years) attending a general medical clinic, 75% had a lifetime diagnosis of major depression compared with 18% of euthyroid subjects (132). Others studies, however, did not confirm this association (23, 88, 126).

Although older individuals could be more susceptible to the effects of a disease for their possible frailty, few studies were specifically designed to assess the effect of sHT on QoL in older population (126, 133). Functional capacity as assessed by ADL and IADL plays a fundamental role in determining QoL of older people. The majority of the studies, however, reported no association between sHT and functional impairment (23, 96, 126, 133, 134). Simonsick et al. documented that individuals aged between 70 and 79 years with mild TSH elevation (4.5–7 mIU/L) had faster mean and usual gait speed and better cardiorespiratory fitness than euthyroid controls (96). In addition, a prospective, observational, population-based study carried out on 599 participants followed up from age 85 to 89 years showed no association between serum TSH and FT4 levels and disability in daily life, depressive symptoms, and cognitive impairment at baseline or during follow-up. Increasing serum TSH levels were associated with a lower mortality rate that remained after adjustments for baseline disability and health status (23). In a very recent population-based prospective study, the Octabaix study, 307 (184 females) inhabitants older than 85 years were submitted to complete multifunctional assessment, including Barthel and Lowton indexes, mini mental state examination (MMSE), mini nutritional assessment (MNA), gait rating scale, and quality of life test (EQ-VAS). Compared to euthyroid subjects (*n* = 280), those with sHT (*n* = 20) did not show any difference either in QoL, or in functional and cognitive status (133).

Overall, these data support the hypothesis that in the oldest old population, individuals with abnormally high levels of TSH do not experience adverse effects and may have a prolonged life span. The studies focused on a specific class of patients (older than 85 years), and the results should be carefully interpreted, also considering the weakness of observational studies. Nonetheless, these data together with the results obtained by Rozing et al. (20) that demonstrated a possible genetic predisposition of nonagenarians to a decrease function of hypothalamus-pituitary-thyroid axis suggest that the oldest old may represent a different population with respect to moderate old people or young adults. In this setting, however, the lack of association between mild hypothyroidism and poor QoL as assessed by SF-36 was observed also in individuals older than 65 years, without known thyroid disease who had participated in the Korean Longitudinal Study on Health and Aging (126). Thus, the observation of a progressive shift of the normal serum TSH range toward higher values from healthy young individuals up to centenarians (7) may indicate a certain degree of down-regulation of the hypothalamus–pituitary–thyroid-peripheral axis and a possible adaptive response to age-related metabolic changes.

## Conclusion

Consistent results among large prospective cohort studies suggest a relationship between sHT and HF progression and events in older people, while an impact of sHT in terms of CHD events and mortality is recognized only in young adults (<65 years). Moreover, in the oldest old population (>85 years) one study suggested that high levels of TSH not only do not exert adverse effects but also may favor a prolonged lifespan. Limited reports are available on QoL of elderly people, and the lack of an association between sHT and poor QoL is generally reported. Thus, moderately old patients (65–75 years) could be considered clinically similar to the adult population, albeit with a higher optimal TSH target value (around 2–3 mIU/l), whereas oldest old subjects (>80–85 years) should be carefully followed with a wait-and-see strategy, generally avoiding hormonal treatment (18, 135).

Large, randomized, prospective studies are nonetheless warranted to assess whether sHT actually affects CV disease progression and events as well as health-related QoL in older people, analyzing results according to the age groups of participants.

## Author Contributions

All the authors substantially contributed to the study for important intellectual content.

## Conflict of Interest Statement

The authors declare that the research was conducted in the absence of any commercial or financial relationships that could be construed as a potential conflict of interest.
